# Subjective cognitive complaints in cognitively unimpaired Parkinson’s disease: Associations with affective burden, motor severity, and awareness-related processes

**DOI:** 10.1007/s10072-026-09300-0

**Published:** 2026-08-12

**Authors:** Henrique Soares Dutra Oliveira, Francisco Eduardo Costa Cardoso, Paulo Caramelli, Sarah Teixeira Camargos

**Affiliations:** 1 Faculdade de Ciências Médicas de Minas Gerais, Faculdade de Medicina, Departamento de Clínica Médica, Belo Horizonte, Minas Gerais Brazil; 2 Faculdade de Medicina, Departamento de Clínica Médica, Universidade Federal de Minas Gerais, Belo Horizonte, Minas Gerais Brazil

**Keywords:** Parkinson’s disease, Subjective cognitive complaints, Cognitively unimpaired, Mood symptoms, Anosognosia

## Abstract

**Background:**

Subjective cognitive complaints (SCC) are frequent in Parkinson’s disease (PD), but their significance in cognitively unimpaired individuals remains uncertain.

**Objectives:**

To investigate the frequency of SCC in cognitively unimpaired PD patients and examine associations with objective cognition, mood symptoms, and anosognosia.

**Methods:**

In this cross-sectional study, 74 PD patients with preserved cognitive performance based on the Addenbrooke’s Cognitive Examination–Revised (ACE-R) were included. SCC were assessed using the Prospective and Retrospective Memory Questionnaire (PRMQ-10). Mood symptoms were measured with the Hospital Anxiety and Depression Scale (HADS). Associations were analyzed using nonparametric tests, correlations, and multivariable linear regression. Anosognosia was estimated using a subjective–objective discrepancy index.

**Results:**

SCC were present in 36 participants (48.6%). SCC status was not associated with ACE-R total or memory scores. SCC+ participants showed higher affective symptom burden and greater motor severity. PRMQ-10 scores correlated with HADS measures, particularly depressive symptoms. In adjusted models, SCC status showed a modest positive association with ACE-R total scores. **No descriptive evidence of systematic discrepancy between subjective and objective memory measures was observed**.

**Conclusions:**

SCC were highly prevalent in cognitively unimpaired PD and were more strongly associated with affective burden and motor severity than with objective cognitive performance.

## Introduction

Cognitive impairment is one of the most frequent and disabling non-motor manifestations of Parkinson’s disease (PD), and it can occur at any stage of the disease course [1–3]. The cognitive spectrum in PD extends from subjective cognitive complaints (SCC) to mild cognitive impairment (MCI) and dementia, with substantial heterogeneity in clinical expression and progression [4–6].

Within this spectrum, SCC have attracted increasing interest as a possible early clinical manifestation of cognitive vulnerability in PD [7, 8]. SCC refer to self-perceived decline in cognition, with or without objective cognitive impairment, and may be present even in cognitively normal individuals [7, 8]. In PD, this construct has gained attention particularly in patients who do not yet meet formal criteria for PD-MCI or dementia [7, 9].

SCC are commonly reported in PD, but reported frequencies vary substantially across studies because of heterogeneity in definitions, instruments, and study populations [8, 10]. A recent systematic review and meta-analysis estimated a pooled SCC prevalence of approximately 36%, while also demonstrating that prevalence is influenced by clinical and methodological factors [10]. In cognitively unimpaired PD, prevalence estimates vary widely [7, 11]. However, the evidence base underlying these estimates is derived predominantly from studies conducted in high-income countries, with only limited representation from middle-income settings and no formal stratification by country or income level, which may constrain the global generalizability of these findings.

The clinical significance of SCC in PD remains uncertain. Cross-sectional studies have demonstrated inconsistent associations between subjective complaints and objective cognitive performance, with some reporting weak or absent relationships [8, 10, 12–14]. Longitudinal evidence is also heterogeneous. While some studies suggest that SCC may predict subsequent cognitive decline, progression to MCI, or functional deterioration, others do not support a consistent prognostic role [10, 13–15].

An alternative explanation is that SCC may reflect non-cognitive factors. A growing body of evidence indicates that subjective complaints are more strongly associated with neuropsychiatric symptoms, including depression, anxiety, and apathy, than with objective cognitive deficits [10, 11]. In addition, individual differences in cognitive reserve may influence the perception and reporting of cognitive difficulties. Educational attainment and life-course cognitive engagement may modulate the relationship between underlying brain changes and clinical expression, potentially affecting how patients interpret and report subjective decline. [15].

Despite increasing interest in SCC in PD, their meaning in cognitively unimpaired individuals remains unclear. In particular, it is uncertain whether SCC reflects subtle objective cognitive dysfunction or is better explained by affective and awareness-related processes. Therefore, the present study aimed to investigate the prevalence of SCC in a well-characterized sample of cognitively unimpaired individuals with PD and to examine their relationship with objective cognitive performance and affective symptoms.

## Methods

### Design, setting, participants, and sampling

This cross-sectional observational study was designed and reported in accordance with the Strengthening the Reporting of Observational Studies in Epidemiology (STROBE) statement guideline [16]. Participants were consecutively recruited between June 2024 and December 2025 from the Movement Disorders Outpatient Clinic of the Neurology Service at the Hospital das Clínicas Universidade Federal de Minas Gerais (HC-UFMG), and from the Neurogeriatrics Outpatient Clinic of the Faculdade de Ciências Médicas de Minas Gerais, Belo Horizonte, Brazil.

Eligible participants were individuals with Parkinson’s disease (PD) diagnosed according to the International Parkinson and Movement Disorder Society (MDS) clinical diagnostic criteria [17, 18], with disease onset greater than or equal to 50 years of age [19], symptom duration ≥ 1 year, preserved communicative ability, and normal cognitive performance according to Brazilian normative data for the Addenbrooke’s Cognitive Examination–Revised (ACE-R) [20]. Cognitive unimpaired status was defined as ACE-R total score within 1.5 standard deviations of age- and education-adjusted Brazilian norms [20] and absence of functional impairment attributable to cognitive decline as assessed with the Pfeffer Functional Activities Questionnaire [21]. The ACE-III, which is very similar to the ACE-R, presented good accuracy in the diagnosis of MCI in a Brazilian sample of PD patients [22].

Exclusion criteria included: exposure to dopamine-blocking drugs; prior neurosurgery; other neurological disorders; structural brain lesions; severe primary psychiatric disorders; traumatic brain injury; metabolic or storage diseases; and regular use of medications with relevant cognitive effects.

Sampling was non-probabilistic and consecutive. All eligible individuals presenting during the study period were invited to participate, following clinic scheduling order. Given the non-random nature of sampling, multivariable models were prespecified to account for potential confounding variables.

### Data collection

All assessments were conducted by the same trained neurologist (HSDO) with expertise in movement disorders and cognitive evaluation to minimize inter-examiner variability. The structured assessment followed a standardized order for all participants.

### Clinical assessment

Demographic and clinical data were collected using a structured case report form, including age, sex, years of schooling, symptoms’ duration, levodopa equivalent daily dose (LEDD) [23], Hoehn and Yahr stage (H&Y) [24], motor severity measured with the MDS-UPDRS part III [25], and vascular comorbidities (hypertension and diabetes). Sleep-related measures included REM sleep behavior disorder assessed with the RBDQ-1 [26], sleep quality assessed with the Sleep Quality Scale (SQS) [27], and daytime sleepiness measured with the Epworth Sleepiness Scale [28]. Gait was evaluated using the Timed Up and Go Test (TUGT) [29]. Psychotropic medications were defined as the use of antidepressants, mood stabilizers, or antipsychotics. Benzodiazepines, anticholinergic agents, and high-dose tricyclic antidepressants were part of the exclusion criteria due to their known cognitive effects.

### Neuropsychological and neuropsychiatric assessments

Global cognition was assessed using the ACE-R. SCC were measured using the Brazilian version of the 10-item Prospective and Retrospective Memory Questionnaire (PRMQ-10) [30]. SCC were operationalized categorically. Participants scoring ≥ 1.5 standard deviations above Brazilian normative means for the PRMQ-10 [30] were classified as SCC-positive (SCC+). Accordingly, PRMQ-10 was treated as a categorical exposure variable in primary analyses. Mood symptoms were assessed using the Hospital Anxiety and Depression Scale (HADS) [31]. Apathy was measured using the Starkstein Apathy Scale [32]. Visual hallucinations were screened using item 4 of the Non-Motor Symptoms Scale (NMSS-4) [33].

Anosognosia was quantified using an index of subjective–objective discrepancy, the impaired self-awareness of cognitive deficits (ISACog) [34]. ISACog was calculated as: ISACog = Z(ACE-R Memory) − Z(PRMQ-10 inverted). PRMQ scores were inverted so that higher values represented better perceived memory function. Standardized z-scores were used to place subjective and objective measures on a common metric. Interpretation followed discrepancy-based frameworks: ISACog < 0 → anosognosia (objective impairment with reduced complaint). ISACog > 0 → over-reporting (subjective complaint disproportionate to objective performance). ISACog ≈ 0 → **relative subjective–objective alignment**. The distribution of ISACog values was examined descriptively to assess the presence of systematic bias toward under- or over-awareness. This approach is consistent with discrepancy methodologies used in metacognitive and awareness research in neurodegenerative conditions [34]. Because both subjective and objective measures were standardized within-sample, the resulting index reflects relative alignment rather than absolute metacognitive accuracy and is not designed to test for group-level bias against an external reference.

### Sample size calculation

The primary outcome was the prevalence of SCC. Based on the 2024 meta-analysis by Siciliano et al. [8], the pooled prevalence of SCC in PD was approximately 35–40%. We hypothesized that prevalence in our cohort might be higher given socioeconomic vulnerability and lower educational attainment, consistent with evidence indicating increased subjective cognitive concerns among minority and socially diverse populations [35]. Assuming an expected prevalence of 50%, a two-sided alpha of 0.05, beta of 0.20 (power 80%), and precision of ± 12%, the minimum required sample size was estimated at 67 participants. All calculations and statistical procedures were conducted using Stata 18 [36].

### Statistical analysis

**Continuous variables were described using medians and interquartile ranges. Categorical variables were presented as frequencies and percentages. Because several continuous variables showed non-normal distributions**,** univariable analyses were performed using Mann–Whitney U tests**,** Spearman rank correlation**,** or chi-square tests**,** as appropriate.** Multivariable linear regression models were constructed to examine the independent association between SCC and objective cognitive performance. Covariates were prespecified based on theoretical relevance and prior literature, including age, years of schooling, disease duration, motor severity (UPDRS III), and mood symptoms (HADS total score). Covariate selection was not based solely on statistical significance in univariable analyses but on clinical plausibility and potential confounding structure. **Before interpreting the regression model**,** assumptions of linear regression were evaluated by assessing residual normality**,** homoscedasticity**,** linearity**,** and multicollinearity**,** and no major violations were identified.** Statistical significance was defined as *p* < 0.05.

## Results

### Baseline characteristics and prevalence of SCC

A total of 74 cognitively unimpaired individuals with PD were included in the analysis. SCC were present in 36 participants (48.6%; 95% CI 36.9–60.6%).

Baseline characteristics according to SCC status are presented in Table 1. The SCC + and SCC− groups were comparable with respect to age, sex distribution, years of schooling, symptoms’ duration, LEDD, and Hoehn & Yahr stage. Vascular comorbidities and use of psychotropic medications did not differ significantly between groups.

**Table 1:** Baseline characteristics according to subjective cognitive complaint status

Variable	SCC− (*n* = 38)	SCC+ (*n* = 36)	*p* value
Sex (Male/Female)	25/13	22/14	0.8098
Age, years	67.5 [63.0–72.0]	66.0 [61.0–70.0]	0.2787
Years of Schooling	11.0 [7.2–15.0]	11.0 [6.8–13.5]	0.8052
Years of Disease	5.5 [3.0–7.8]	5.0 [3.0–6.2]	0.7726
LEDD (mg)	600.0 [400.0–1000.0]	575.0 [300.0–737.5]	0.2310
H&Y	2.0 [2.0–2.0]	2.0 [2.0–2.0]	1.0000
UPDRS-Motor	16.5 [12.0–28.0]	26.0 [19.5–32.5]	**0.0464**
Hypertension	20 (53%)	11 (31%)	0.0636
Diabetes	8 (21%)	6 (17%)	0.7690
Psychotropic Medications	14 (37%)	16 (44%)	0.6364
RBDQ-1	25 (66%)	25 (69%)	0.8067
SQS	8.0 [6.2–9.0]	6.0 [5.0–8.0]	**0.0118**
NMSS-4	0 (0%)	3 (8%)	0.1101
Epworth	5.5 [3.0–7.0]	8.0 [5.0–11.2]	**0.0145**
Apathy	8 (21%)	15 (42%)	0.0788
TUGT	10.9 [9.5–13.7]	10.2 [9.3–12.4]	0.1523
HADS-T	5.0 [3.0–7.8]	11.5 [7.8–17.2]	0.000046*
HADS-A	3.0 [1.0–5.0]	6.0 [3.0–10.0]	0.0007*
HADS-D	1.0 [1.0–3.0]	6.5 [3.0–8.2]	0.000003*
MMSE	29.0 [28.0–29.0]	29.0 [27.8–29.2]	0.6633
ACE-R Total Score	84.5 [81.0–91.8]	88.5 [83.5–92.2]	0.2488
ACE-R (Attention and Orientation)	17.0 [17.0–18.0]	17.0 [16.0–18.0]	0.7684
ACE-R (Memory)	19.0 [16.2–21.8]	19.5 [18.0–25.0]	0.2985
ACE-R (Verbal Fluency)	11.0 [10.0–13.0]	11.0 [11.0–13.0]	0.4143
ACE-R (Language)	24.5 [23.0–26.0]	25.0 [24.0–25.0]	0.7399
ACE-R (Visuospatial Abilities)	15.0 [14.0–15.0]	15.0 [14.0–16.0]	0.5758

Continuous and ordinal variables are presented as median [IQR]. Categorical variables are presented as n (%). Sex is presented as Male/Female counts. *P* values were calculated using Mann–Whitney U tests for continuous/ordinal variables and Fisher’s exact tests for categorical variables. Two-tailed tests were used

*ACE*-*R* Addenbrooke’s Cognitive Examination–Revised, *CI* confidence interval, *Epworth* Epworth Sleepiness Scale, *H&Y* Hoehn & Yahr stage, *HADS-A* Hospital Anxiety and Depression Scale – Anxiety subscore, *HADS-D* Hospital Anxiety and Depression Scale – Depression subscore, *HADS-T* Hospital Anxiety and Depression Scale – Total score, *LEDD* Levodopa equivalent daily dose, *MMSE* Mini-Mental State Examination, *NMSS-4* Non-Motor Symptoms Scale, hallucinations subdomain, *RBDQ-1* REM Behavior Disorder Screening single question, *SCC* subjective cognitive complaints, *SQS* Sleep Quality Scale, *TUGT* Timed Up and Go test, *UPDRS-Motor* Unified Parkinson’s Disease Rating Scale – Motor section

(1 A) Comparison of ACE-R Total scores between participants without (SCC−) and with (SCC+) subjective cognitive complaints using the Mann–Whitney U test. No significant difference was observed (U = 577.0, z = − 1.153, *p* = 0.249, *r* = 0.134). (1 B) Comparison of ACE-R Memory subdomain scores between SCC groups. No significant difference was detected (U = 588.0, z = − 1.040, *p* = 0.299, *r* = 0.121). (1 C) Correlation between PRMQ-10 Total score (higher scores indicate worse perceived functioning) and ACE-R Total score. Spearman correlation revealed a non-significant association (ρ = 0.205, *p* = 0.080). The regression line and 95% confidence band are displayed for visualization purposes

(2 A) Comparison of HADS-Total scores between SCC − and SCC+ groups using the Mann–Whitney U test. SCC+ participants showed significantly higher mood symptom burden (U = 307.5, z = − 4.075, *p* < 0.001, *r* = 0.474). (2B) Correlation between PRMQ-10 Total score and HADS-Total score. A moderate positive association was observed (Spearman ρ = 0.457, *p* < 0.001), indicating that worse perceived cognitive functioning is associated with greater mood symptom severity

Motor severity was higher in the SCC+ group (median UPDRS-Motor 26.0 vs. 16.5; *p* = 0.046). Sleep-related measures also differed, with lower sleep quality (SQS, *p* = 0.012) and higher daytime sleepiness (Epworth, *p* = 0.015) among SCC+ participants. Mood measures showed the most pronounced between-group differences. The SCC+ group had significantly higher HADS total scores (median 11.5 vs. 5.0; *p* < 0.001), as well as higher anxiety and depression subscores (both *p* < 0.001). Apathy showed a higher occurrence, although it was not statistically significant, in the SCC+ group (*p* = 0.078). Global cognitive performance did not differ significantly between groups (ACE-R Total: *p* = 0.249; MMSE: *p* = 0.663), nor did any ACE-R subdomain scores (all *p* > 0.29).

### SCC and objective cognitive performance

**Fig. 1:**
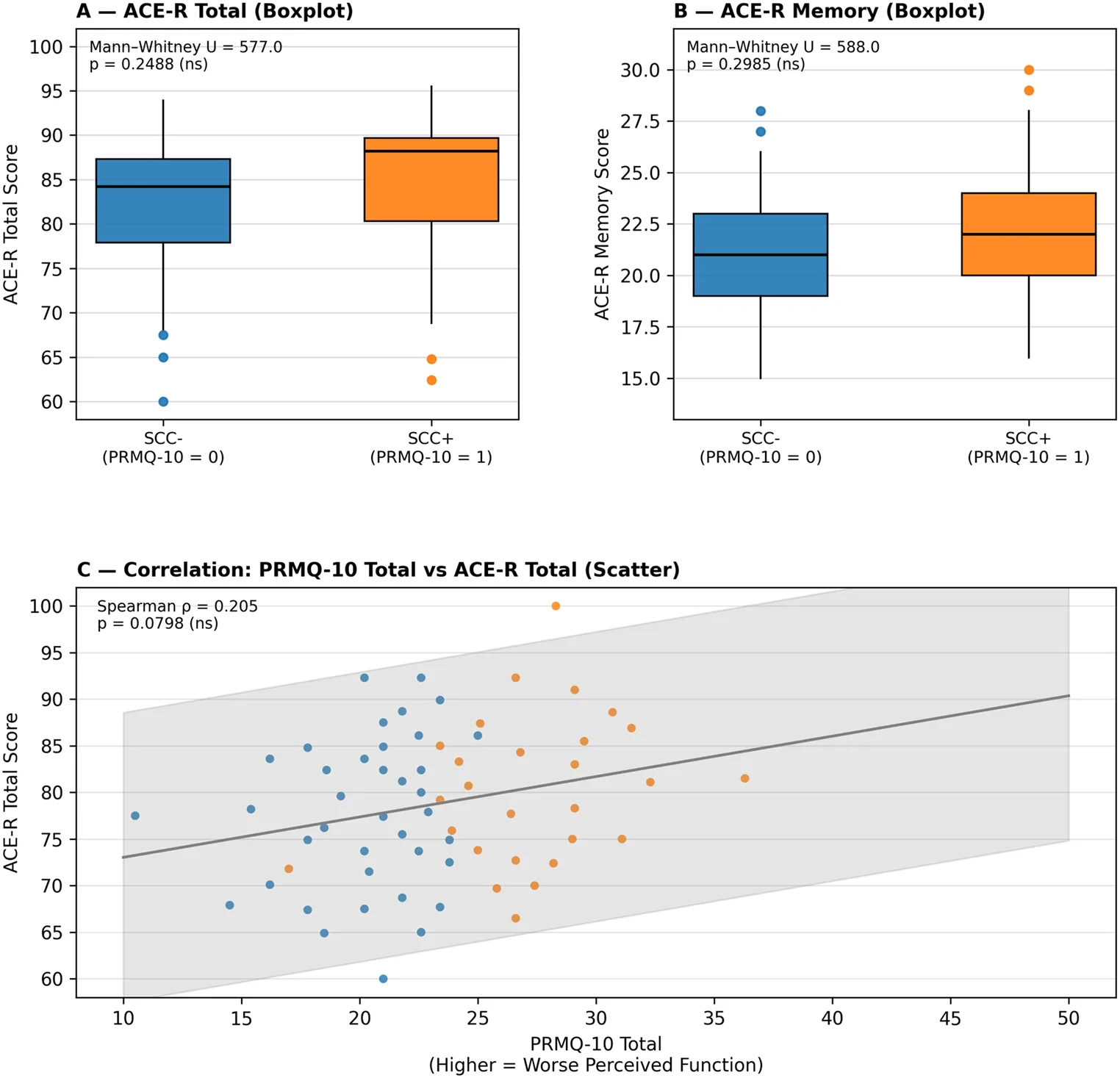
Association between subjective cognitive complaints and objective cognitive performance

There was no significant difference in ACE-R Total scores between SCC − and SCC+ participants (Mann–Whitney U = 577.0; *p* = 0.2488) (Fig. 1A). Similarly, ACE-R Memory subdomain scores did not differ between groups (U = 588.0; *p* = 0.2985) (Fig. 1B). When PRMQ-10 total score was analyzed as a continuous variable, the correlation with ACE-R Total score was not statistically significant (Spearman ρ = 0.205; *p* = 0.0798) (Fig. 1C).

### Mood symptoms and SCC

**Fig. 2:**
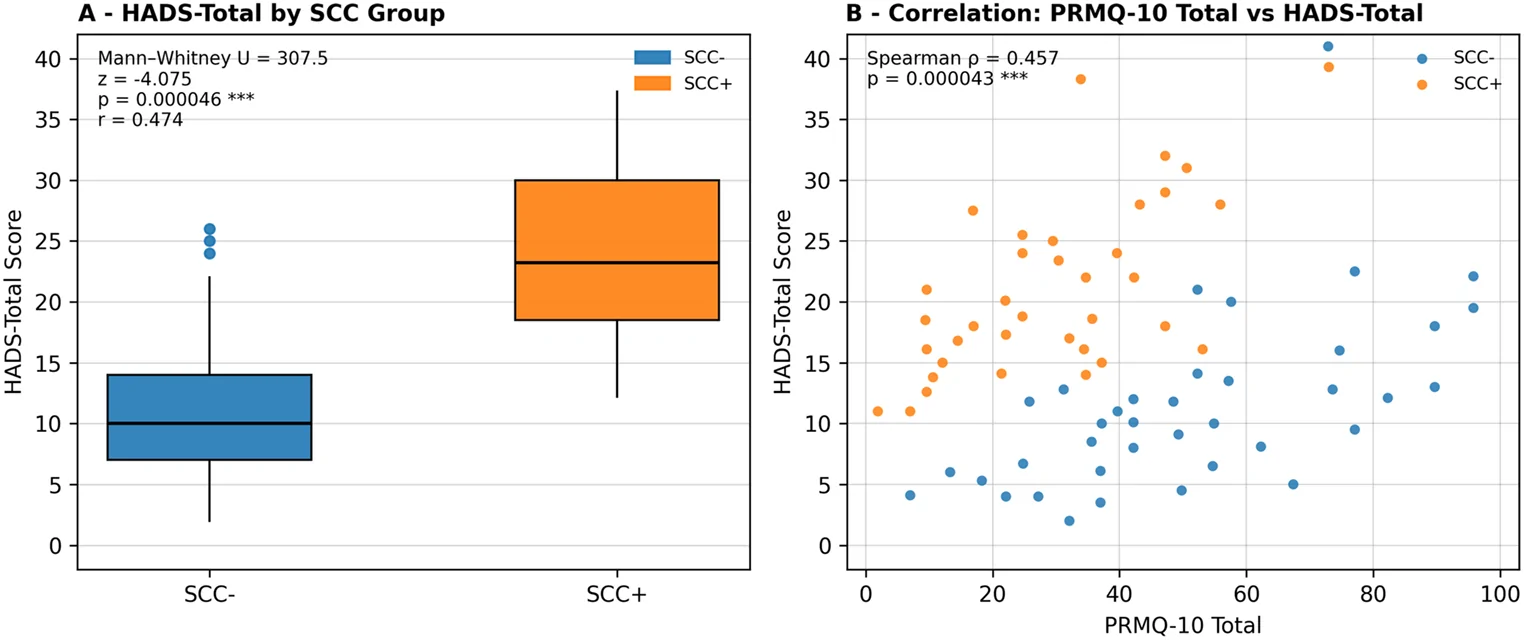
Mood contribution to subjective cognitive complaints

Participants with SCC + had higher mood symptom burden. HADS total scores were significantly higher in the SCC+ group (U = 307.5; *p* < 0.001) (Fig. 2A). PRMQ-10 total score correlated with HADS total score (Spearman ρ = 0.457; *p* < 0.001), indicating an association between greater SCC and higher mood-symptom severity (Fig. 2B).

### Multivariable analysis of SCC and ACE-R scores

In multivariable linear regression adjusting for age, years of schooling, symptoms’ duration, motor severity (UPDRS-Motor), and HADS total score, SCC status was independently associated with ACE-R Total score (β = 2.95; 95% CI 0.03–5.88; *p* = 0.048). The model explained 40% of the variance in ACE-R Total score (adjusted R² = 0.40).

### Anosognosia

The mean ISACog value was 0.00 (SD = 1.52; 95% CI − 0.35 to 0.35). A one-sample test against zero showed no significant deviation (*p* = 1.00). However, because both components of the awareness index were standardized within-sample, the mean-centered result reflects the scaling approach and does not allow inference about group-level bias toward anosognosia or over-reporting.

## Discussion

In this sample of cognitively unimpaired individuals with PD, SCC were frequent but were not consistently associated with global cognitive performance as measured by ACE-R in unadjusted analyses, with only a modest association observed in adjusted models, in which SCC+ status was associated with slightly higher ACE-R scores. Instead, the most consistent signal was affective burden, with the SCC+ group showing higher levels of anxiety, depression, and overall neuropsychiatric symptoms, together with a higher proportion of apathy.

The prevalence observed in our sample is higher than the pooled estimate reported in the recent meta-analysis (36%) [8], but remains within the range described in prior studies [9–11, 37–40]. Importantly, prevalence estimates are highly dependent on methodological factors. Studies restricted to cognitively unimpaired individuals tend to report higher frequencies of SCC, and variability is also influenced by the instrument used to define complaints and by sample characteristics such as disease severity and demographic profile [8, 38, 39, 41–43]. In this context, our estimate likely reflects both the inclusion of cognitively normal patients and the use of a memory-weighted instrument. **This distinction deserves consideration. Although SCC represent a multidomain construct**,** the current PD literature recognizes that most available assessment instruments predominantly capture memory-related complaints**,** reflecting the absence of standardized multidomain assessment instruments. In our study**,** the PRMQ-10 predominantly assesses prospective and retrospective memory complaints; therefore**,** our findings primarily reflect the memory-related component of the broader SCC construct.**

The lack of a consistent cross-sectional association between SCC and ACE-R–based cognitive performance is consistent with a large body of literature. The recent meta-analysis demonstrated only weak and negligible associations between SCC and objective cognition [8]. Similarly, systematic reviews and observational studies have reported inconsistent or absent relationships, particularly in cognitively unimpaired cohorts [2, 39, 40, 42, 44–47]. Studies such as Purri et al. [9] and Koster et al. [40] showed that subjective complaints may be present despite preserved global cognition, reinforcing the notion that SCC in this context do not reliably reflect measurable impairment. However, because ACE-R was used both as a screening and outcome measure, restriction of range may have attenuated detectable associations, particularly in this cognitively unimpaired sample. Additionally, ACE-R is a global screening instrument and may underrepresent executive and prospective memory processes more directly captured by PRMQ-10, which may have limited the ability to detect associations in the domains most relevant to subjective complaints.

In contrast, the association between SCC and affective symptoms is robust and consistently supported across studies. Chua et al. [36] demonstrated that SCC in early PD with normal cognition were associated with affective symptoms, and similar findings have been reported in multiple cohorts showing strong relationships with depression and anxiety [3, 11, 37, 46, 48, 49]. Barbosa et al. [3] framed this relationship as a central question in PD—whether complaints reflect mood disturbance or early cognitive decline—highlighting that affective symptoms are a major driver of subjective perception. This interpretation is further supported by evidence outside PD, where subjective cognitive perception is more closely related to mood than to objective performance [50]. Taken together, these findings support a strong association between SCC and affective burden in cognitively unimpaired PD. However, the directionality of this relationship cannot be determined in this cross-sectional design. Longitudinal studies are needed to clarify the directionality of these associations.

The higher proportion of apathy observed in the SCC+ group, although not statistically significant, is consistent with this broader affective profile. Apathy has been identified as a relevant correlate of SCC in PD, including in systematic reviews [49, 51], and has been linked to alterations in motivation and goal-directed behavior that may influence subjective perception of cognitive efficiency. Additionally, studies examining self-awareness in PD have shown that subjective reporting is influenced by both mood and insight [41, 52, 53]. Therefore, the observed pattern likely reflects a multidimensional affective-behavioral construct rather than isolated depressive or anxious symptoms.

In addition to affective and behavioral factors, motor severity may also contribute to the expression of subjective cognitive complaints at the individual level. In our sample, the SCC+ group presented higher UPDRS motor scores, a difference that is clinically meaningful and suggests a greater overall disease burden. Notably, a recent meta-analysis by Siciliano et al. [8] reported that UPDRS Part III scores did not significantly moderate SCC prevalence across studies, indicating that motor severity may not explain differences in prevalence at the population level. However, this does not preclude an association at the individual level within specific samples.

In this context, our findings suggest that, although motor severity may not determine whether SCC are present across different study populations, it may influence how patients perceive and report cognitive difficulties within a given disease context. Patients with more severe motor symptoms may experience increased effort in daily activities, fatigue, and reduced efficiency, which could generalize to a perception of cognitive decline. Therefore, SCC may reflect an integrated perception of disease burden, encompassing motor, affective, and cognitive dimensions, rather than isolated cognitive impairment. The apparent contradiction between higher ACE-R scores in SCC+ patients in adjusted analysis and their greater affective burden requires careful interpretation. This pattern suggests that the adjusted association may reflect differences in awareness or reporting rather than true differences in cognitive capacity. Rather than indicating a true paradox, this finding may reflect the interaction between preserved cognitive function and symptom awareness. Individuals with better cognitive performance may retain the capacity to detect subtle inefficiencies in daily functioning, while affective symptoms increase the salience and perceived impact of these changes. Conversely, reduced awareness has been described in PD, particularly in association with lower depressive symptoms and frontal dysfunction [53]. This bidirectional model—greater reporting with preserved cognition and potential underreporting with reduced awareness—offers a coherent explanation for our findings.

**The inclusion of a metacognitive awareness framework adds an additional dimension to the interpretation of our results. By quantifying the discrepancy between subjective perception and objective performance**,** we explored subjective–objective alignment rather than formally testing awareness. The absence of a systematic discrepancy in the ISACog distribution provides descriptive evidence of relative alignment between subjective and objective memory measures at the group level. However**,** because both components were standardized within-sample**,** this finding should be interpreted cautiously and should not be considered evidence for preserved awareness or against anosognosia. Instead**,** the ISACog index should be viewed primarily as an indicator of internal consistency between subjective and objective measures rather than a measure of absolute metacognitive accuracy.**

Other clinical variables further support this interpretation. The SCC+ group presented with higher motor severity and worse sleep-related measures, including sleep quality and daytime sleepiness. These factors may contribute indirectly to subjective cognitive inefficiency through mechanisms such as fatigue, attentional fluctuations, and reduced cognitive stamina. Previous studies have shown that SCC in PD are associated with broader non-motor symptom burden and may vary according to disease characteristics and subtypes [43, 54].

The prognostic value of SCC remains uncertain. While some longitudinal studies suggest that SCC may predict future cognitive decline [13, 14, 43, 44, 55, 56], others have failed to demonstrate a consistent association [57]. The meta-analysis also highlights this heterogeneity, with limited and conflicting longitudinal evidence [8]. Thus, although SCC may warrant closer clinical monitoring, they cannot be considered reliable predictors of cognitive decline in cognitively unimpaired PD. Some limitations should be considered. The absence of biomarkers limits the ability to detect underlying neurodegenerative changes. **The PRMQ-10 primarily assesses prospective and retrospective memory complaints and therefore may underrepresent subjective difficulties involving executive**,** visuospatial**,** language**,** and attentional functions. Consequently**,** although we retained the broader concept of SCC**,** our findings should be interpreted as reflecting the memory-related component of this construct. Future studies incorporating multidomain subjective cognitive instruments may help determine whether these associations extend beyond that.**

The adjusted association between SCC and higher ACE-R scores should be interpreted cautiously, as it may reflect model dependence or suppression effects related to covariates such as mood and sleep, rather than a true positive relationship. Additionally, because ACE-R was used both for participant selection and as an outcome measure, restriction of range may have reduced sensitivity to detect associations between subjective complaints and objective cognitive performance.

Another point is that objective cognition was assessed using a global screening instrument, which may not capture subtle domain-specific deficits characteristic of PD, particularly in executive and visuospatial functions. Furthermore, there may be a mismatch between the constructs assessed by PRMQ-10, which emphasizes prospective and executive aspects of memory, and the ACE-R endpoints used in this study, which may be less sensitive to these processes. Future studies using domain-specific neuropsychological measures may further clarify whether subjective complaints relate to executive, attentional, or visuospatial functions not captured by global screening tools.

Overall, our findings suggest that, in cognitively unimpaired PD, SCC appear to be more strongly associated with affective symptoms and greater motor severity than with ACE-R–based cognitive performance and may even be associated with slightly higher performance in adjusted analyses in this cognitively unimpaired sample. Their clinical relevance may therefore lie less in identifying early cognitive decline in cross-sectional assessment and more in signaling patients with increased neuropsychiatric vulnerability.

## Conclusions

SCC are common in cognitively unimpaired individuals with PD but are not consistently associated with objective cognitive performance as measured by global screening instruments such as the ACE-R. Instead, SCC appear to reflect a multidimensional construct integrating affective burden, motor severity, and **awareness-related processes**, suggesting that they may represent a subjective marker of overall disease impact rather than an early indicator of objective cognitive decline in cross-sectional assessment. From a clinical perspective, this supports the need for a comprehensive evaluation of patients presenting with SCC, beyond cognitive screening alone, while their potential prognostic value warrants further investigation in longitudinal studies using domain-specific cognitive measures.

## Data Availability

The raw data supporting the conclusions of this article will be made available by the authors, without undue reservation.
